# Supplementary figures and images for: The Genetic Mechanisms Underlying the Concerted Expression of the yellow and tan Genes in Complex Patterns on the Abdomen and Wings of Drosophila guttifera
Source: Genes (Basel). 2023 Jan 24;14(2):304. doi: 10.3390/genes14020304 (PMC9957387; doi:10.3390/genes14020304)

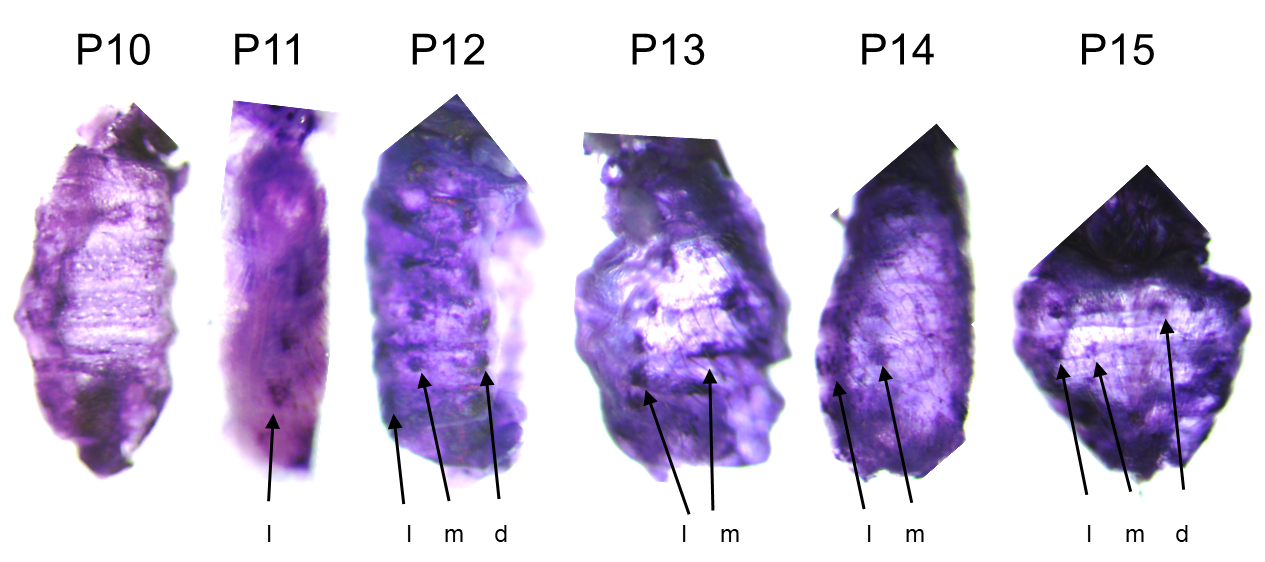

Supplement: Supplementary file 1 [file genes-14-00304-s001.zip › Figure S1.tif]

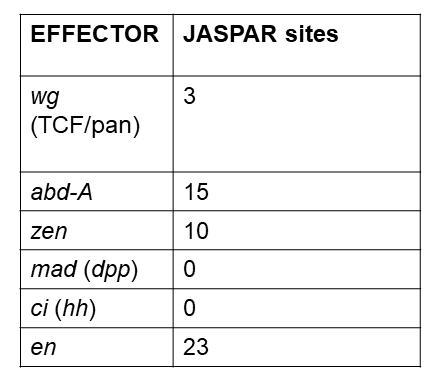

Supplement: Supplementary file 1 [file genes-14-00304-s001.zip › Table S1.TIF]
